# Who does the model learn from?

**DOI:** 10.1016/S2589-7500(21)00057-1

**Published:** 2021-04-12

**Authors:** Marie-Laure Charpignon, Leo Anthony Celi, Mathew Cherian Samuel

**Affiliations:** Institute for Data, Systems, and Society, Massachusetts Institute of Technology, Cambridge, MA 02139, USA; MIT Critical Data, Massachusetts Institute of Technology, Cambridge, MA 02139, USA; MIT Critical Data, Massachusetts Institute of Technology, Cambridge, MA 02139, USA; Department of Medicine, Beth Israel Deaconess Medical Center, Boston, MA, USA; Department of Biostatistics, Harvard T H Chan School of Public Health, Boston, MA, USA; MIT Critical Data, Massachusetts Institute of Technology, Cambridge, MA 02139, USA

Despite restrictions on surgeries and procedures during the COVID-19 pandemic in the USA, 2020 saw 8906 liver transplantations—more than in any previous year.^1^ Liver transplant recipients often face risks of complications, such as graft failure, infection, cancer, or cardiovascular disease, and experience elevated mortality rates. For clinicians responsible for patient follow-up, monitoring, and preventative care, being able to predict and anticipate these complications is valuable.

In *The Lancet Digital Health*, Osvald Nitski and colleagues provide a machine learning method both to identify patients at increased risk for one of these complications and to predict the magnitude of that risk, 1 year and 5 years after transplantation.^2^ Building upon recent literature on deep neural networks for survival analysis,^3^ they applied a Transformer model to the prediction of post-operative outcomes among patients from two distinct cohorts in North America. Rather than relying on a select set of human-curated variables, deep learning models are able to process the full patient medical history, identify temporal trends, and automatically uncover non-linear relationships in the raw data. Such machine-learned patterns can be missed by domain experts and remain uncaptured by heuristic-driven model formulation.

Nitski and colleagues initially trained their deep learning predictive risk scoring system on data from 42 146 adult patients who were transplanted between 2002 and 2014 and were part of the large US Scientific Registry of Transplant Recipients (SRTR); the authors then fine-tuned the model and tested it on the regional University Health Network (UHN) dataset of 3269 adult patients from a single large transplant programme in Ontario, Canada, transplanted between 1986 and 2014. The investigators showed that the model successfully predicted transplant-related mortality (ie, death due to graft failure, infection, cancer, or cardiovascular disease) within 5 years of transplantation, suggesting that it could be used to provide continuously updated mortality risk scores to guide clinical decision making during follow-up. The model’s excellent performance across these two patient samples from vastly different healthcare settings suggests robustness and transferability. Notably, this methodology differs from previous work by incorporating a greater number of predictors (190 input variables in the SRTR dataset and 63 in the UHN dataset) and allowing the model to learn more complex relationships in a data-driven manner. Additionally, the authors addressed model explainability,^4^ a recognised barrier to the adoption of machine learning models in health care, by using SHAP values—a concept borrowed from game theory—to determine sociodemographic factors (eg, recipient age at transplantation) and clinical factors (eg, hepatocellular carcinoma) influencing the predicted risk scores over multiple time horizons. This step is not only crucial in interpreting the rationale behind the predictions, but also a key safety check for flawed assumptions and specifications during modelling.

Despite this comprehensive approach, a fundamental question is who does the model learn from? An implicit assumption when a model is trained on data is that the study cohort represents the target population for the algorithm. If this assumption is incorrect, the analysis might lead to spurious associations. For example, in a recent observational study among US veterans with SARS-CoV-2 infection,^5^ the authors reported that expected risk factors such as smoking and obesity were not associated with COVID-19 mortality. These observations are contradictory to published literature.^6,7^ This artifact results from a bias in the sample selection that distorts the known association in the general population. In this instance, the patient’s likelihood of being hospitalised was conditioned on the risk factors of interest, smoking and obesity. Indeed, obesity prevalence^8^ and reported smoking rates among US veterans^9^ have consistently been higher than in the civilian population, and most COVID-19 clinical studies consist solely of tested patients requiring hospital admission. Collider bias is a byproduct of such restricted analysis: any detected associations would not reflect individual causal effects, neither within the study sample nor in the broader population.

Collider bias could similarly affect prediction modelling of post-transplant outcomes. In Nitski and colleagues’ study, the model was trained and validated on patients who underwent liver transplantation in North America. Notably, while the US SRTR dataset includes relevant demographic information about race and ethnicity of transplant recipients, the Ontario-based UHN does not provide such data, thus making it challenging to assess differences in pre-transplant and post-transplant care between population subgroups. However, both in North America and globally, equal access to liver transplantation remains challenging for racial and ethnic minorities. Three major sources of disparities appear along a transplant patient’s journey: from examination by a primary care provider, to referral to a specialist, to selection for transplantation, to awaiting a donor, to successful transplantation. First, a patient with liver disease who was not referred to a transplant centre upon presentation to their primary care provider would never be represented in the dataset used for training and might thus respond differently. Even after adjusting for comorbidities, organ disease stage, and type of health insurance coverage, multiple studies reveal physician biases in listing practices that disproportionately affect certain regions.^10^ Second, geographical discrepancies in waiting times and transplantation rates result in some groups being more likely to die before transplantation. Third, informational, social, and financial support in the continuum of care after transplantation influences adherence, a major driver of graft survival, and disproportionately impacts vulnerable populations.

With an increasing number of organ transplantations every year, the application of deep learning offers tremendous possibilities to enhance the prediction of transplantation outcomes and tailor the delivery of post-surgery preventative care. Nitski and colleagues propose a promising approach, leveraging recent advances in deep learning, that could be applied to the prediction of complications from kidney, lung, or heart transplantation. Importantly, the predicted risk scores produced by such algorithms are ultimately provided to clinicians to inform care management decisions. For these risk indices to effectively individualise post-transplantation interventions going forwards, the data science and medical communities together need to critically assess the extent to which underlying biases in data collection might induce spurious associations and correct for them when possible. Without a careful and systematic evaluation of who the model learns from, such bias will persist and adversely impact the performance of predictive algorithms in practice and, ultimately, patient outcomes.
